# Global mangrove soil organic carbon stocks dataset at 30 m resolution for the year 2020 based on spatiotemporal predictive machine learning

**DOI:** 10.1016/j.dib.2023.109621

**Published:** 2023-09-26

**Authors:** Tania L. Maxwell, Tomislav Hengl, Leandro L. Parente, Robert Minarik, Thomas A. Worthington, Pete Bunting, Lindsey S. Smart, Mark D. Spalding, Emily Landis

**Affiliations:** a Conservation Science Group, Department of Zoology, University of Cambridge, Cambridge, UK; b EnvirometriX Ltd, Wageningen 6708 PW, the Netherlands; c OpenGeoHub foundation, Wageningen 6708 PW, the Netherlands; d Department of Geography and Earth Sciences, Aberystwyth University, Aberystwyth, SY23 3DB, UK; e The Nature Conservancy, Arlington, VA, USA; f The Nature Conservancy, Strada delle Tolfe, 14, Siena, 53100, Italy

**Keywords:** Blue carbon, Carbon sequestration, Coastal ecosystem, Spatial modelling, Mangroves

## Abstract

This dataset presents global soil organic carbon stocks in mangrove forests at 30 m resolution, predicted for 2020. We used spatiotemporal ensemble machine learning to produce predictions of soil organic carbon content and bulk density (BD) to 1 m soil depth, which were then aggregated to calculate soil organic carbon stocks. This was done by using training data points of both SOC (%) and BD in mangroves from a global dataset and from recently published studies, and globally consistent predictive covariate layers. A total of 10,331 soil samples were validated to have SOC (%) measurements and were used for predictive soil mapping. We used time-series remote sensing data specific to time periods when the training data were sampled, as well as long-term (static) layers to train an ensemble of machine learning model. Ensemble models were used to improve performance, robustness and unbiasedness as opposed to just using one learner. In addition, we performed spatial cross-validation by using spatial blocking of training data points to assess model performance. We predicted SOC stocks for the 2020 time period and applied them to a 2020 mangrove extent map, presenting both mean predictions and prediction intervals to represent the uncertainty around our predictions. Predictions are available for download under CC-BY license from 10.5281/zenodo.7729491 and also as Cloud-Optimized GeoTIFFs (global mosaics).

Specifications Table

**Table:** 

Subject	Agricultural Sciences (Soil Science), Environmental Science, Computer Science (Computer Science Applications)
Specific subject area	Soil carbon in mangroves, remote sensing signal processing, spatiotemporal machine-learning modeling
Type of data	Raster data (TIF files) Code files
How the data were acquired	Training data was compiled from published sources USGS Earth Resources Observation and Science (EROS): Analysis Ready Data Landsat bands (Blue, Green, Red, NIR, SWIR1, SWIR2) Climatologies at high resolution for the earth's land surface areas (CHELSEA): precipitation, mean, min. and max. air temperature NASA Moderate Resolution Imaging Spectroradiometer (MODIS): land surface temperature and enhanced vegetation index MERIT digital elevation model: elevation EC JRC/Google: global surface water probability
Data format	Processed
Description of data collection	Training data were based on a previous dataset [1], and recent publications [2], [3], [4], [5], [6]. For predictions, we used a number of covariate layers: • Time-series 2000–2020: ARD Landsat bands [7], derived vegetation indices, CHELSA images (precipitation, mean, min. and max. air temperature [8]), MODIS LST (1km) and EVI (250m) • Static layers: MERIT DEM elevation [9], global surface water probability [10], long-term climatic variables, global composites of Landsat bands [11]
Data source location	Global, using a recent 2020 mangrove extent map [12]. This represents a total mangrove extent of 147,359 km^2^ ranging from 39 degrees South to 33 degrees North ARD Landsat bands: https://glad.umd.edu/ard/home CHELSEA images: https://chelsa-climate.org/ MODIS LST: https://modis.gsfc.nasa.gov/data/dataprod/mod11.php MODIS EVI: https://modis.gsfc.nasa.gov/data/dataprod/mod13.php MERIT DEM: http://hydro.iis.u-tokyo.ac.jp/∼yamadai/MERIT_Hydro/ Global surface water: https://global-surface-water.appspot.com/ Long-term climatic variables and global composites of Landsat bands: https://storage.googleapis.com/earthenginepartners-hansen/GFC-2022-v1.10/download.html
Data accessibility	The predicted soil organic carbon maps at 30m resolution and their upper and lower prediction intervals can be found in the following repository [13]: Repository name: Zenodo Data identification number: 10.5281/zenodo.7729492 Direct URL to data: https://doi.org/10.5281/zenodo.7729491
	Detailed code associated with the data analysis is available from the Github repository https://github.com/OpenGeoHub/spatial-prediction-eml/, which is archived in the following repository [14]: Repository name: Zenodo Data identification number: 10.5281/zenodo.5894924 Direct URL to data: https://zenodo.org/record/5894924

## 1 Value of the Data

• The map provides global soil organic carbon stock estimates for mangroves, using refined statistical methods such as spatiotemporal ensemble machine learning
• The map can support research on changes in soil organic carbon stocks over time, can guide restoration and protection efforts, and can be used to inform Nationally Determined Contributions as defined by the Paris Agreement under the United Nations Framework Convention on Climate Change (UNFCCC). It can also be used to compare soil organic carbon stocks between different coastal typologies, marine ecoregions of the world, or other administrative units (i.e. countries, protected areas, etc.)
• The methodology and code can be reproduced to calculate soil organic carbon stocks in other ecosystems or local scale analyses

## 2 Objective

The main objective of this dataset was to improve the previously produced map of soil organic carbon (SOC) in mangroves at 30m resolution [1] by using more training data points, mapping to an updated mangrove 2020 extent layer [12] instead of the 2000 extent layer, and implementing improved statistical methods. More specifically, we used spatiotemporal (time-series images + long-term layers + soil depth as predictors) Ensemble Machine Learning (EML). We selected EML as it is less prone to overfitting and extrapolation problems, as opposed to using one learner such as Random Forest. We modeled SOC content (%) and bulk density separately, which were then aggregated to SOC density and to fixed depths. Additionally, we used spatial cross-validation instead of random cross-validation methods, as this has been shown to more accurately assess models’ predictive performance in spatial modeling.

## 3 Data Description

Predictions are provided in the “mangroves_tiles_SOC_predictions_2020.zip” folder in a tiled format. Each tile is named according to its geographic location (i.e. 089E_21N corresponds to 89E to 90E, 21N to 22N). The “tile_mangroves_typology_v3_modis_sinu.gpkg” file contains the tile locations, and the “mangroves_typology_v3_cog.tif” file contains the mangrove extent into which predictions were made [12].

The data presented in each tile are maps of predicted soil organic carbon (%), bulk density (g cm^-3^), and soil organic carbon stocks (tonnes per hectare, hereafter referred to as megagrams C per hectare) in mangroves at 30 m resolution, predicted for the soil horizon 0–100 cm (Table 1). There are three stock maps, which are GeoTIFF raster files: the mean prediction, the lower prediction interval and the upper prediction interval, to indicate modeling uncertainty around predicted values. We estimated prediction intervals using the 95 % probability lower and upper ranges.

**Table 1:** Files located in each tile of the “mangroves_tiles_SOC_predictions_2020.zip” folder, corresponding to global maps of SOC in mangroves to 1m depth at 30m resolution, for the most recent predicted time period (2020-2021).

File description	File name
Predicted SOC content (%) for 0–100 cm	sol_soc.wpct_mangroves.typology_m_30m_s0..100cm_2020_global_v1.1.tif
Predicted bulk density (g cm-3) for 0–100cm	sol_db.od_mangroves.typology_m_30m_s0..100cm_2020_global_v0.1.tif
Predicted mean SOC stocks (Mg ha-1) for 0–100 cm	sol_soc.tha_mangroves.typology_m_30m_s0..100cm_2020_global_v0.1.tif
Lower 95% probability prediction interval of predicted SOC stocks (Mg ha-1) for 0–100 cm	sol_soc.tha_mangroves.typology_l.std_30m_s0..100cm_2020_global_v0.1.tif
Upper 95% probability prediction interval of predicted SOC stocks (Mg ha-1) for 0–100 cm	sol_soc.tha_mangroves.typology_u.std_30m_s0..100cm_2020_global_v0.1.tif

Detailed code associated with the data analysis is available from the Github repository (https://github.com/OpenGeoHub/spatial-prediction-eml/), allowing for predictions to be reproduced. The corresponding code file for this analysis “spatiotemporal-soc.Rmd” is located in the main Github repository folder.

## 4 Experimental Design, Materials and Methods

### 4.1 Training data

**Fig. 1:**
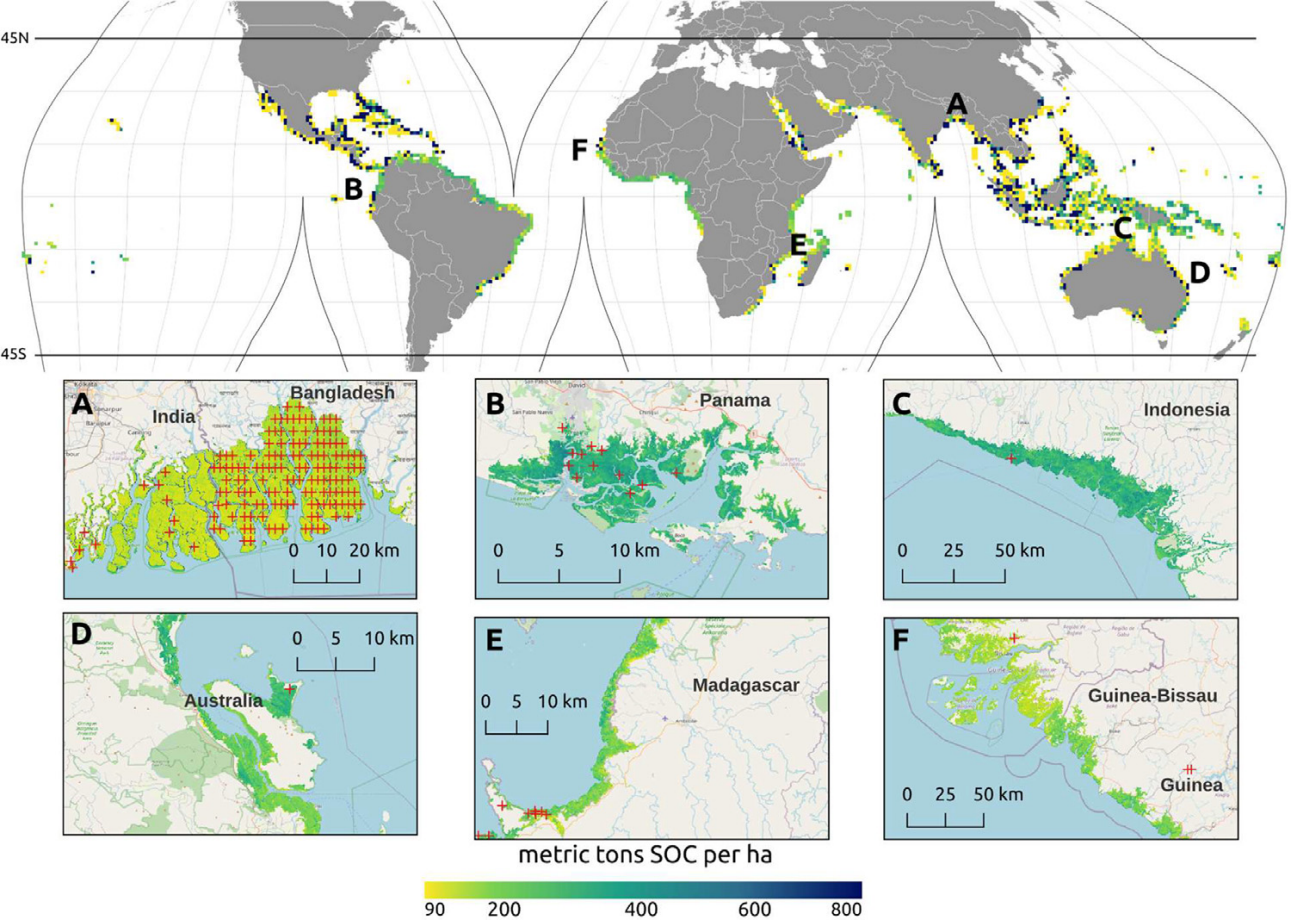
Global distribution of mangrove soil organic carbon stocks (metric tons SOC per hectare) predicted in 2020 for the top meter of soil (pixel ∼ 10 000 km^2^), and detailed maps (30 m resolution) for selected regions of the world: (A) Sundarbans along the India/Bangladesh border, (B) Bahía de los Muertos, Pacific coast of Panama, (C) southwest coast of Papua, Indonesia, (D) Hinchinbrook Island, Queensland, Australia, (E) Ambaro Bay, Madagascar, and (F) Guinea-Bissau and Guinea along the West African coast. In the top panel, data presented as mean stock (Mg C ha^−1^) for mangrove forest area only within each pixel. In the bottom panel, red crosses represent training data from both mangrove sampling and from complementary sources used to help map the transition zones.

**Fig. 2:**
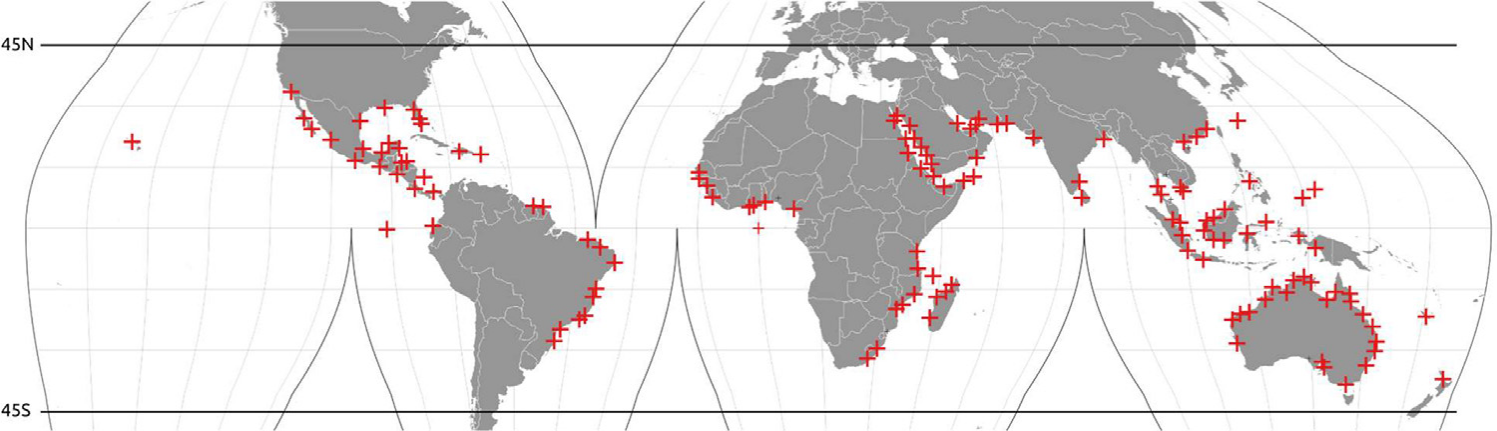
Global distribution of mangrove training data points from all sources falling into mangrove tiles, used to model SOC. From all samples, a total of 10,331 samples (3299 unique locations) had measurements of SOC.

We used a compilation of soil samples analyzed in the laboratory and digitized primarily from peer-reviewed literature. The original set from Sanderman et al. 2018 [15] was extended with additional samples collated from more recent literature sources [2], [3], [4], [5], [6]. We also incorporated some points in non-mangrove areas, to help model transition zones from mangroves to non-mangrove areas (Fig. 1) (Fig. 2).

### 4.2 Spatial modeling of soil organic carbon stocks

To produce a reliable estimate of global SOC stock in mangroves and also to map their distribution, we used spatiotemporal EML [14]. We used an approach where SOC (g kg^−1^) and BD were predicted independently as a function of depth (*d*) and spatially explicit temporal and static covariate layers (X_p_), then aggregated to derive SOC stocks [16]:

$$\begin{array}{ccc} OC_{\text{pred}}\left[g\;kg^{-1}\right] & = & d+X_{1}\left(x,y\right)+X_{2}\left(x,y\right)+....X_{p}\left(x,y\right)\\ BD_{\text{pred}}\left[g\;cm^{-3}\right] & = & d+X_{1}\left(x,y\right)+X_{2}\left(x,y\right)+....X_{p}\left(x,y\right)\\ \text{SOC}\;\text{stock}\;\text{Mg}\;ha^{-1} & = & OC_{\text{pred}}\;\left[g\;kg^{-1}\right]\;{\;}^{*}10\;\;{\;}^{*}\;BD_{\text{pred}}\;\left[g\;cm^{-3}\right]\;{\;}^{*}\text{horizon}\;\text{thickness}\;\left[\text{cm}\right]{\;}^{*}\\ & & 100\;\left[\text{Mg}\;ha^{-1}/g\;cm^{-2}\right] \end{array}$$

where xyd are the 3D coordinates: latitude and longitude in decimal degrees and soil depth (measured to the center of a horizon). By including depth in the model, this avoided the need to extrapolate training points to a 1 m depth.

**Fig. 3:**
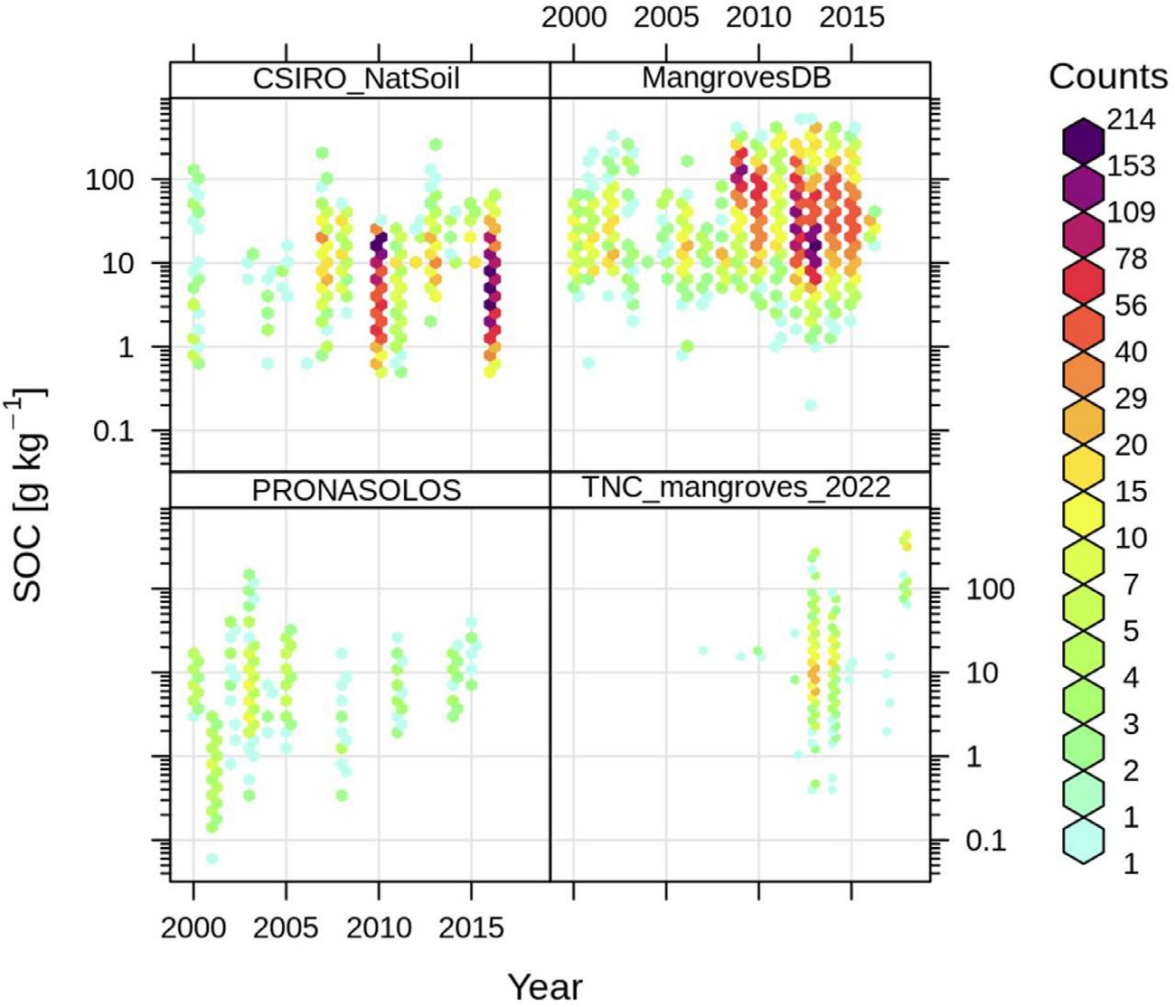
Distribution of training points through time. This figure compares SOC content data in mangroves from the mangrove database [1] and the recently compiled data [2], [3], [4], [5], [6], as well as data used to help model the transition zones (CSIRO_NatSoil from [17], and PRONASOLOS from [18]).

To integrate time for the spatiotemporal modeling, we divided the training data points into five time periods (2002 = 2000–2003, 2006 = 2004–2007, 2010 = 2008–2011, 2014 = 2012–2015, 2018 = 2016–2019, 2020 = 2020–2021), and used time-series from these periods for the predictive modeling, along with the same long-term (static) variables for all periods. Thus, the model is trained using data points from all time periods and their corresponding time-series data, improving overall accuracy for the most recent 2020 soil carbon map presented here. We see from Fig. 3 that there are enough points spread over time for spatiotemporal mapping of SOC.

Finally, we used EML by combining predictions from three learners using the mlr R package [19]. For EML the modeling algorithm becomes secondary, so that the final model is less prone to overfitting and extrapolation problems, as opposed to using one learner such as a Random Forest.

### 4.3 Covariate layers

The spatially explicit temporal and static covariate layers (X_p_) we used to predict soil organic carbon include:

• Globally consistent time-series 2000–2020 ARD Landsat bands (Blue, Green, Red, NIR, SWIR1, SWIR2) [7], aggregated and gap-filled to produce complete consistent lower quantiles (P25 = lower 0.25 probability) [9],
• Time-series of CHELSA images representing climate precipitation, mean, minimum and maximum air temperature [8],
• MODIS LST (1km) and EVI (250m) monthly time-series (covering 2000–2020 period) generated using aggregation,
• Number of static (long-term) layers including MERIT DEM elevation [9], global surface water probability [10], long-term climatic variables, and global composites of Landsat bands from 2010, 2014 and 2018 [11].

In addition to original Landsat bands, we also used the Landsat Enhanced Vegetation Index (EVI) that can be derived from Landsat data. The Landsat bands and derivatives are available at 30-m spatial resolution, while the 250m and 1km resolution images had to be downscaled to 30-m spatial resolution (here we used GDAL and cubic-spline downscaling).

### 4.4 Model validation

**Fig. 4:**
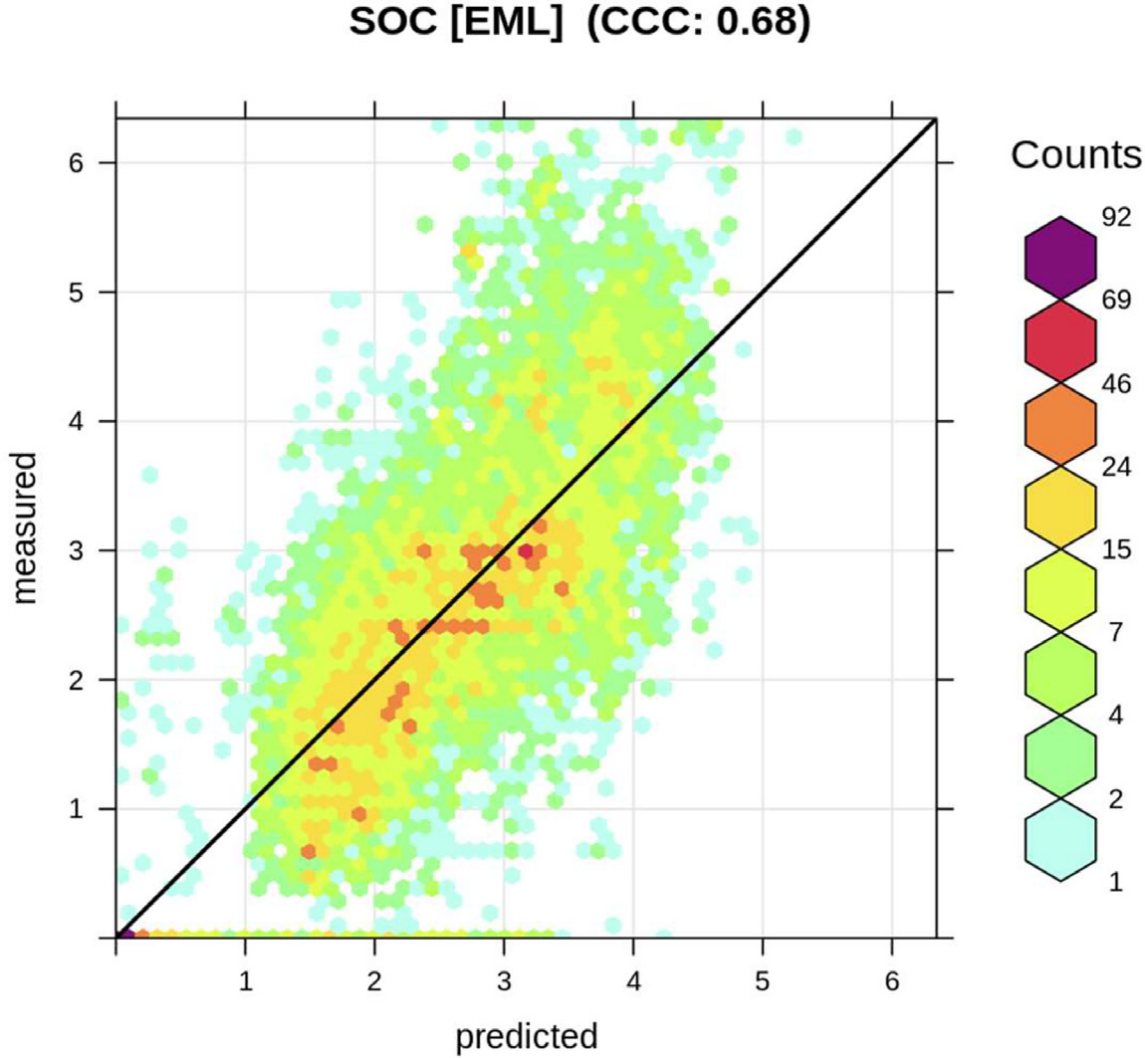
Accuracy plot for soil organic carbon fitted using Ensemble Machine Learning (EML).

**Fig. 5:**
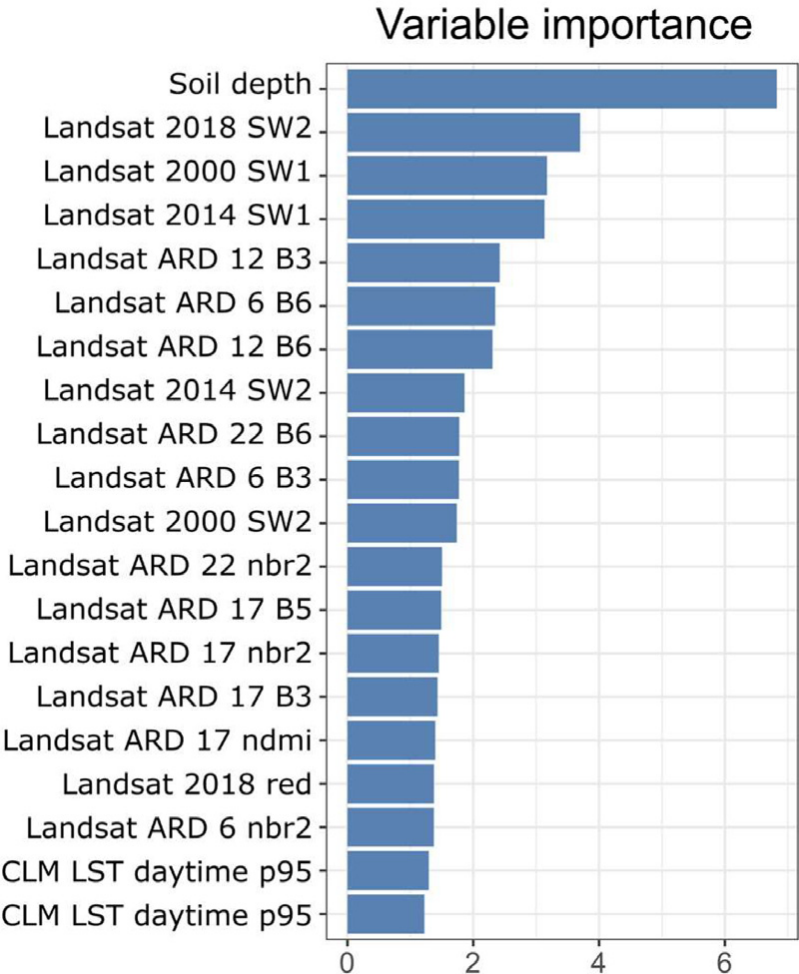
Variable importance for 3D prediction model for SOC based on random forest. SW2 = Short wave infrared, ARD = analysis ready data [7], B*N* = band number *N*, nbr2 = Normalized Burn Ratio 2, ndmi = Normalized Difference Moisture Index, CLM LST daytime p95 = climate land surface temperature of the 95th quantile probability of daytime. Processing of the Landsat time-series of images is described in [20].

To account for spatial clustering of training data points in the model cross-validation, we validated the machine learning models using spatial blocks so that a subset of points was either used for training or cross-validation (CV). To do so, we used the mlr R package [19] and a spatial block ID. This led to a drop of the R-squared of the model, from 0.82 (using random CV) to 0.44 (using spatial CV), but reduced overfitting the training points (Figs. 4 and 5).

### 4.5 Producing predictions of SOC and BD

Once we fitted independent models for SOC and BD, we generated predictions for all time-periods and for standard depths (0, 30, 60, 100 cm), within the 2020 global mangrove extent map at 30 m resolution [12]. We aggregated these predictions to calculate SOC stocks for the horizon 0-100 cm. The maps in this dataset include the mean predictions, as well as the lower prediction interval and the upper prediction interval, to indicate modeling uncertainty around predicted values. We used two standard deviations to estimate prediction intervals so these are the 95 % probability intervals.

Based on spatiotemporal prediction of SOC stocks, we estimated that the global SOC stocks for world mangrove forests in 2020 are, on average, about 350 MgC/ha for 0–100 cm depth (67 % prob. interval: 232–470 MgC/ha) i.e. about 4.6 gigatonnes (67 % prob. interval: 3.1–6.2).

## Ethics Statements

The authors declare that the hereby presented data and data article fully comply with the Journal's policy in terms of authors’ duties, data integrity, and experimental requirements.

## CRediT authorship contribution statement

**Tania L. Maxwell:** Writing – original draft, Data curation. **Tomislav Hengl:** Data curation, Methodology, Software, Validation, Visualization, Writing – review & editing. **Leandro L. Parente:** Data curation, Methodology, Software, Validation. **Robert Minarik:** Visualization, Writing – review & editing. **Thomas A. Worthington:** Writing – review & editing. **Pete Bunting:** Methodology, Writing – review & editing. **Lindsey S. Smart:** Data curation, Writing – review & editing. **Mark D. Spalding:** Supervision, Writing – review & editing. **Emily Landis:** Supervision, Funding acquisition, Writing – review & editing.

## Data Availability

Global mangrove soil carbon data set at 30 m resolution for year 2020 (0-100 cm) (Original data) (Zenodo).
